# Ring Wrinkle Patterns with Continuously Changing Wavelength Produced Using a Controlled-Gradient Light Field

**DOI:** 10.3390/ma11091571

**Published:** 2018-09-01

**Authors:** Hongye Li, Bin Sheng, He Wu, Yuanshen Huang, Dawei Zhang, Songlin Zhuang

**Affiliations:** Engineering Research Center of Optical Instruments and Systems, Ministry of Education and Shanghai Key Laboratory of Modern Optical Systems, University of Shanghai for Science and Technology, Shanghai 200093, China; 167710556@st.usst.edu.cn (H.L.); 18818226501@163.com (H.W.); hyshyq@sina.com (Y.H.); dwzhang@usst.edu.cn (D.Z.); slzhuang@yahoo.com (S.Z.)

**Keywords:** gradient wrinkles, UV/ozone, irradiance

## Abstract

We report a facile method to prepare gradient wrinkles using a controlled-gradient light field. Because of the gradient distance between the ultraviolet (UV) lamp and polydimethylsiloxane (PDMS) substrate during UV/ozone treatment, the irradiance reaching the substrate continuously changed, which was transferred into the resulting SiO*_x_* film with a varying thickness. Therefore, wrinkles with continuously changing wavelength were fabricated using this approach. It was found that the wrinkle wavelength decreased as the distance increased. We fabricated 1-D wrinkle patterns and ring wrinkles with a gradient wavelength. The ring wrinkles were prepared using radial stresses, which were achieved by pulling the center of a freely hanging PDMS film. The resulting wrinkles with changing wavelength can be used in fluid handling systems, biological templates, and optical devices.

## 1. Introduction

Wrinkling and buckling are common phenomena in nature, e.g., surface patterns of plants, skin wrinkles, and mountain ranges [1,2,3]. Surface wrinkling has recently attracted attention as a useful method to fabricate micro- and nanostructures because it is facile, cost effective, and does not use conventional lithography [4,5,6,7]. The most commonly used model for surface wrinkling is a bilayer membrane composed of a rigid, thin elastic surface layer on top of a soft, thick elastic substrate. When depositing a metal film, or conducting oxygen or ultraviolet (UV)/ozone (O_3_) treatment of a pre-stretched polydimethylsiloxane (PDMS) substrate, sinusoidal wrinkles are formed after release of the pre-strain because of the strain mismatch between layers with different elastic moduli [8,9,10].

Many applications of wrinkles have been reported, such as tunable diffraction gratings, microlenses, mechanical property measurement of thin films, and microfluidic channels based on anisotropic wetting [4,11,12,13,14,15]. In these applications, wrinkle patterns with different feature sizes exhibit different surface properties such as transmittance and anisotropic wetting characteristics. Therefore, there is a demand for various wrinkle groove structures with different feature sizes. There have been many attempts to fabricate gradient wrinkle patterns [16,17,18]. Claussen et al. [19] presented a method to continuously change the elastic modulus of PDMS. Yu and co-workers achieved different symmetry breaking of the wrinkle patterns caused by the tunable thickness gradient in metal films deposited on soft elastic substrates [20]. Lee et al. [21] generated a stepwise gradient wrinkle pattern on a PDMS substrate in which the wavelength could be spatially controlled in each distinct region with a clear boundary.

In this paper, we report a new facile method to fabricate wrinkles with continuously changing wavelength based on a controlled gradient light field. We inclined the PDMS substrate to fabricate gradient 1-D wrinkle patterns. The period of wrinkles is indirectly controlled by simply changing the distance between the UV lamp and PDMS substrate. Based on the conclusion of this experiment, we exposed a PDMS substrate to UV/O_3_ using a cone to provide radial stretch, which in turn enables the thickness of the SiO*_x_* thin film along the radial distribution to be controlled. Then we obtained variable-period ring wrinkles.

## 2. Experimental Section

### 2.1. Materials

The PDMS we used was Sylgard 184 of DOW Corning (Dow Corning, Midland, TX, USA), which consisted of two parts: one was the silicone elastomer; the other was the curing agent. Silicone elastomer was mixed with the curing agent at a 10:1 weight ratio [22], cured at 100 °C for 2 h, and then naturally cooled in air. The cured PDMS with a thickness of 0.5 mm was cut into a circular substrate with a diameter of 20 mm. The PDMS substrate was exposed to UV/O_3_ using a UV lamp (low-pressure mercury lamp, BHK, Claremont, NH, USA) that emitted 185- and 254-nm radiation in atmospheric oxygen (O_2_).

### 2.2. Film Fabrication

The film samples were prepared by UV/O_3_ treatment at room temperature in the presence of atmosphere oxygen [23]. The 185-nm radiation from the lamp produced O_3_, while the 254-nm radiation decomposed the O_3_ into O_2_ and atomic oxygen (O). The atomic oxygen was the chief reactant with PDMS. The oxidation was initiated at the PDMS surface and gradually penetrated into the PDMS. Oxidation converted the organic portion of PDMS to carbon dioxide, water, and some volatile organic compounds that escaped from the PDMS. In contrast, the silicon components did not form volatile compounds under these conditions, thus forming a residual hard layer of SiO*_x_*. The exposure time was 80 min. The film thickness was easily tuned by changing the distance between the lamp and PDMS substrate. To fabricate SiO*_x_* thin films with a thickness gradient, the PDMS substrate was held on an incline or stretched by cones with different angles.

### 2.3. Characterization

The surface morphologies of the samples were observed by an optical microscope (10XB-PC, Shanghai Optical Instrument Factory, Shanghai, China) equipped with a charge-coupled device camera. The profiles of the wrinkle structures were scanned by a white light interferometer (Contour GT-KO, Bruker, Tucson, AZ, USA).

## 3. Results and Discussion

### 3.1. Fabrication of Gradient 1-D Wrinkle Patterns and Sample Characterization 

The wrinkle wavelength *λ* of a buckled bilayer system can be calculated for high strains according to Equation (1) [24]:

(1) $$\lambda =\frac{2\pi h_{f}}{\left(1+\varepsilon_{pre}\right){\left(1+\delta \right)}^{\frac{1}{3}}}{\left[\frac{E_{f}\left(1-\nu_{s}{}^{2}\right)}{3E_{s}\left(1-\nu_{f}{}^{2}\right)}\right]}^{\frac{1}{3}},$$

where $\delta =\frac{5}{32}\left[\varepsilon_{pre}\left(1+\varepsilon_{pre}\right)\right]$ represents the large deformation and geometrical nonlinearity in the substrate, *h_f_* is the thickness of the SiO*_x_* film, ε*_pre_* is the prestrain, and *E* and *ν* are the Young’s modulus and Poisson’s ratio of the substrate (*s*) and film (*f*), respectively. Therefore, *λ* can be controlled by changing *h_f_*, ε*_pre_*, or *E_s_*. In our experiment, *λ* was tuned by changing the thickness of the SiO*_x_* film exposed to the UV/O_3_ treatment. It is well known that in UV/O_3_ treatment, O_3_ is not the primary reactant; instead atomic oxygen produced by the photodissociation of O_3_, reacts with PDMS to form the SiO*_x_* film. Therefore, the thickness of the SiO*_x_* film is related to the concentration of atomic oxygen, which is influenced by the irradiance (*E*). In our experiments, the UV lamp was a line source. Therefore, *E* can be expressed as follows:

(2) $$E=\frac{d\Phi }{dA}=\frac{I}{d}\cos\theta ,$$

where dΦ is the radiation flux on facet dA, and *I* is the radiation intensity of the radiation source. *θ* is the angle between the facet and radiation source, and *d* represents the distance between the illuminated surface and radiation source. The thickness of the SiO*_x_* film and the concentration of the atomic oxygen have a logarithmic relationship [25]. Therefore, the thickness of the SiO*_x_* film can be tuned by *E*. According to Equation (1), $\lambda \propto h_{f}$. Thus, at a fixed radiation intensity and the same exposure time, the continuously changing *λ* can be easily tuned by controlling *d*.

**Figure 1:**
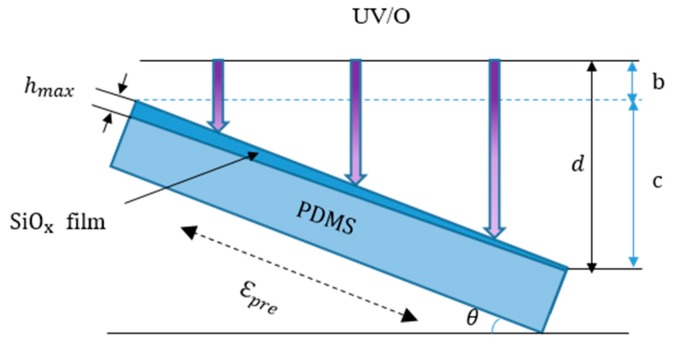
Schematic view of the cross section of a film with a thickness gradient prepared by inclining the PDMS substrate during exposure.

The SiO*_x_* film with a thickness gradient was prepared by inclining the PDMS substrate during UV/O_3_ exposure, as shown in Figure 1. The UV lamp was uniformly scanned by the stepper motor to illuminate the substrate. The sinusoidal wrinkling of the sample with a continuously changing λ was fabricated by exposing an inclined PDMS substrate subjected to 20% pre-strain to UV/O_3_ treatment for 80 min. At a fixed exposure time, when *d* was smaller, the irradiance was greater and the film was thicker. As a result, a film with a thickness gradient formed on the inclined PDMS substrate. The thickness gradient reflected the slope of the film and strongly depended on the dip angle *θ*.

**Figure 2:**
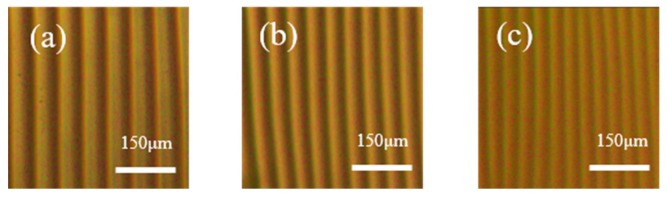
Optical images showing the profiles of wrinkle patterns with different wavelengths at sample positions of (**a**) 0.6 mm, (**b**) 3.0 mm and (**c**) 4.5 mm. Corresponding distances between the lamp and PDMS substrate *d* were (**a**) 7.83 mm, (**b**) 9.15 mm and (**c**) 9.98 mm.

To investigate the wrinkle surface of the thickness-gradient film in more detail, corresponding optical images of the wrinkles at different positions were collected, as presented in Figure 2. Sample position (a) corresponds to *h_max_.* It was found that *λ* decreased significantly from (a) to (c) as *d* increased. Because of the uniform *λ* at each measuring spot, these optical images were representative of the topography at the specific sample positions.

**Figure 3:**
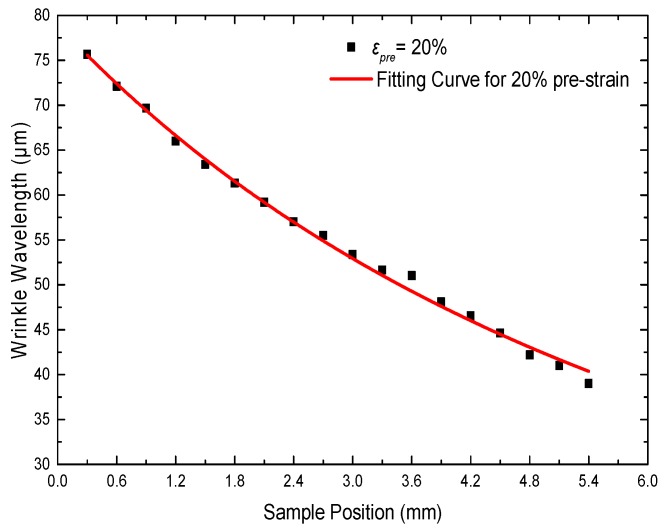
Wrinkle wavelength *λ* as a function of sample position at 20% pre-strain. The red curve represents the fit according to the relation between *λ* and the distance *d* using Equations (3) and (4).

Figure 3 plots *λ* as a function of the sample position. *λ* changed continuously from 75 to 39 μm as the sample position changed. *λ* at different positions was obtained by a white light interferometer. The experimental values showed that the gradient *λ* could be attributed to *d*. We proposed the following correlation between the wavelength and some variables.

(3) $$k=\frac{2\pi }{\left(1+\varepsilon_{pre}\right){\left(1+\delta \right)}^{\frac{1}{3}}}{\left[\frac{E_{f}\left(1-\nu_{s}{}^{2}\right)}{3E_{s}\left(1-\nu_{f}{}^{2}\right)}\right]}^{\frac{1}{3}},$$

(4) $$\lambda \sim k\cdot \mathrm{lg}\left(\frac{I}{d}\cos\theta \right)=k\cdot \mathrm{lg}\left(\frac{I}{x\sin\theta +b}\cos\theta \right),$$

where *k* is related to ε*_pre_*, *x* is the sample position, *I* is the radiation intensity of radiation source, and *d*, *θ*, and *b* are shown in Figure 1. The data for *λ* showed excellent agreement with the prediction of Equations (3) and (4). Therefore, we realized the modulation of *λ* by controlling the irradiance.

### 3.2. Variable-Period Ring Wrinkles Fabrication and Characterization

**Figure 4:**
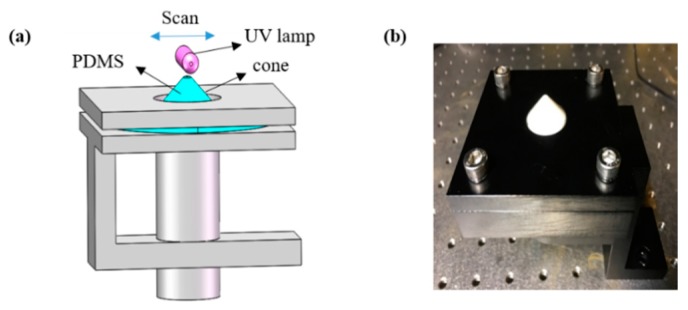
(**a**) Schematic diagram of the experimental device used to prepare ring wrinkles with a continuously changing wavelength. The PDMS membrane was fixed between two plates with round holes and then stretched by a Teflon cone. The UV lamp evenly scanned the substrate. (**b**) Photograph of the experimental setup.

From the 1-D stretching experiment, we know that the wrinkles were perpendicular to the direction of compressive stress. Based on this principle, we produced ring wrinkles using radial stresses [26,27]. In our experiment, ring wrinkles were achieved by pulling the center of a freely hanging PDMS film, as shown in Figure 4. In the pulling device, a Teflon cone with a semi-spheroidal tip pushed the center of the PDMS film from below to stretch the film. We moved the cone by rotating a screw underneath it, so that the cone itself did not rotate to avoid friction with the PDMS film and decrease unwanted strain in the azimuthal direction. The cone was made of Teflon to lower the friction between the cone and PDMS film.

**Figure 5:**
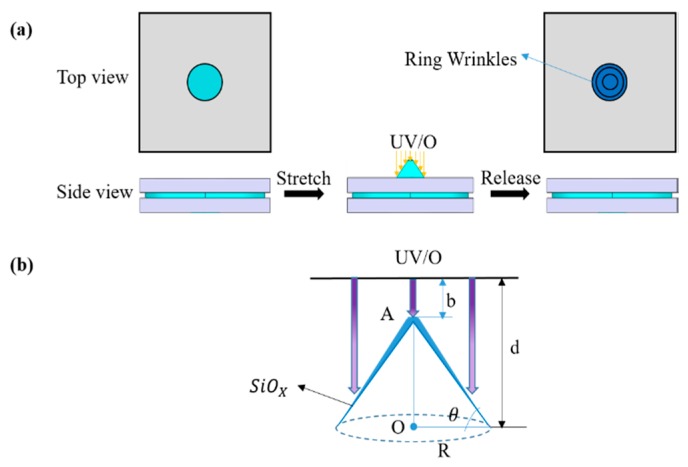
Schematic illustration of the production of concentric ring wrinkles with continuously changing wavelength. (**a**) The center of the substrate was extended upward using a cone and simultaneously treated with UV/O_3_. Concentric wrinkles with continuously changing wavelength were generated after the removal of the cone. (**b**) The thickness gradient of the film was controlled by the distance between the lamp and PDMS substrate *d*.

The ring wrinkle fabrication procedure is shown in Figure 5a. First, the PDMS membrane was fixed between two plates with round holes. The PDMS membrane was 0.5 mm thick. Then the Teflon cone pushed the center of the PDMS membrane. The radius of the stretched membrane was 10 mm. The stretched PDMS substrate was then evenly exposed to UV/O_3_. Ring wrinkles were generated after releasing the pre-strain. According to the theory behind the gradient 1-D wrinkle pattern formation, a SiO*_x_* film with a thickness gradient could be generated in this experiment (Figure 5b). Therefore, variable-period ring wrinkles could be fabricated by this process. The approximate stretching ratio (*SR*) can be calculated using Equation (5),

(5) $$SR=\frac{\sqrt{OA^{2}+R^{2}}}{R},$$

where the *OA* is the height of the cone and *R* is the effective radius of the PDMS membrane (Figure 5b).

**Figure 6:**
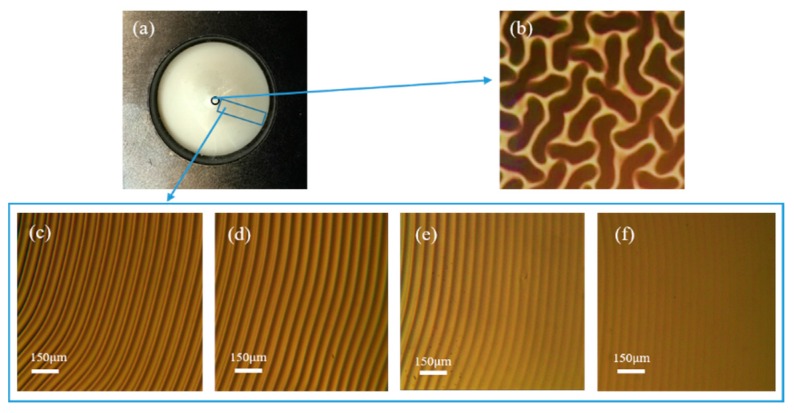
After releasing the pre-strain, the resulting ring wrinkles were observed by an optical microscope. (**a**) The whole membrane in ambient light. (**b**) Optical image of the center of the membrane. (**c**–**f**) Concentric ring wrinkles with continuously changing wavelength from the center of the membrane to the edge in the blue rectangle of Figure 6a corresponding to the gradual increase of *d*.

The ring wrinkles with continuously changing *λ* were fabricated by exposing UV/O_3_ for 80 min on a PDMS substrate subjected to 20% pre-strain generated by the cone. As shown in the optical micrograph in Figure 6b, nonuniformly oriented patterns in the central region were produced because of the friction between the membrane and semi-spheroidal tip of the cone. The size of this region is related to the radian of the cone. Ideally, if the top of a cone does not have a radian, it should avoid the formation of disordered wrinkles in the central region. In the other regions of the membrane, there was no stretching in the circumferential direction, and the strain was directly determined by the percentage of radial stretching. Similar to the 1-D stretching experiment presented above, the distance from the center of the membrane pushed by the cone to the edge *d* gradually increased. Figure 6c–f reveals that *λ* decreased from 102 to 69 μm with increasing *d*.

**Figure 7:**
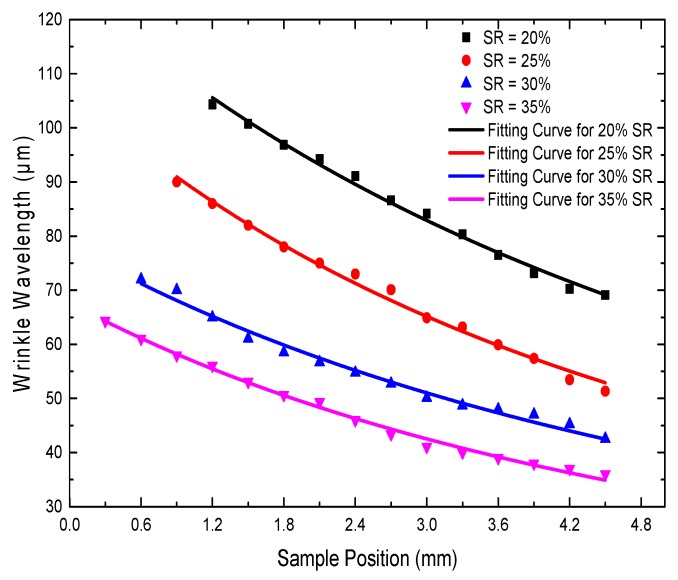
Continuously changing wrinkle wavelengths obtained at different *SR* are schematically depicted as a function of the sample position. The solid lines correspond to the fits according to Equations (3) and (4).

To explore the effect of different pre-strains on *λ* of the resulting wrinkles, we stretched the PDMS membrane by the cone with different *SR*. The UV/O_3_ exposure time was fixed at 80 min. The distance between the lamp and the top of the PDMS membrane was the same in all samples. The *λ* values of the samples were measured by a white light interferometer starting from the boundary of the disordered center of each sample. The measured results are presented in Figure 7. The data for *λ* showed excellent agreement with the prediction of Equations (3) and (4). At each pre-strain, the experimental values showed that *λ* was influenced by the distance *d* between the lamp and the PDMS substrate; that is, *λ* gradually decreased as *d* increased. Figure 7 reveals that for different SR, *λ* decreased as *SR* increased at the same sample position. The larger *θ* corresponded to the larger pre-strain shown in Figure 5b. According to Equations (3) and (4), *λ* decreased as the pre-strain increased. In the same way, *λ* decreased as *θ* increased. Thus, at the same sample position, larger pre-strain resulted in smaller *λ*.

## 4. Conclusions

In summary, we developed a facile method to produce gradient wrinkles using a gradient light field. The continuously changing distance between the lamp and PDMS substrate influenced the irradiance during UV/O_3_ treatment, which then resulted in SiO*_x_* films with varying thicknesses. Therefore, wrinkles with continuously changing *λ* can be fabricated using this approach. We fabricated gradient 1-D wrinkle patterns using a gradient light field. In addition, we also prepared variable-period ring wrinkles by using a cone to stretch the PDMS membrane. Our results demonstrated that the gradient wavelength of wrinkles can be easily controlled by modulating the distance between the lamp and PDMS substrate. The experimental technique presented in this paper can be further developed to effectively control pattern formation, which may be beneficial to provide patterned surfaces for use in optical devices, diffraction gratings, hydrophobicity, fluid handling systems, and biological templates.
